# The development of a novel AND logic based fluorescence probe for the detection of peroxynitrite and GSH†
†Electronic supplementary information (ESI) available: Additional figures, experimental section and original spectra of new compounds. See DOI: 10.1039/c8sc00733k


**DOI:** 10.1039/c8sc00733k

**Published:** 2018-03-16

**Authors:** Adam C. Sedgwick, Hai-Hao Han, Jordan E. Gardiner, Steven D. Bull, Xiao-Peng He, Tony D. James

**Affiliations:** a Department of Chemistry , University of Bath , Bath , BA2 7AY , UK . Email: a.c.sedgwick@bath.ac.uk ; Email: s.d.bull@bath.ac.uk ; Email: t.d.james@bath.ac.uk; b Key Laboratory for Advanced Materials and Joint International Research Laboratory of Precision Chemistry and Molecular Engineering , Feringa Nobel Prize Scientist Joint Research Center , School of Chemistry and Molecular Engineering , East China University of Science and Technology , 130 Meilong Rd. , Shanghai 200237 , China . Email: xphe@ecust.edu.cn

## Abstract

We have developed a novel AND logic based fluorescence probe for the simultaneous detection of ONOO^–^ and GSH (**GSH-PF3**). The **GSH-PF3** probe was synthesised over three steps starting from commercially available fluorescein. The probe was constructed by attaching the GSH reactive motif, 2,4-dinitrobenzenesulfonyl, to the previously reported boronate fluorescence based probe, **PF3**. **GSH-PF3** produced only a small fluorescence response towards the addition of GSH or ONOO^–^ separately. However, when the probe was exposed to both analytes, there was a significant (40-fold) fluorescence enhancement. **GSH-PF3** demonstrated an excellent selectivity towards both GSH and ONOO^–^. In cellular imaging experiments the probe was shown to be cell permeable with no ‘turn-on’ response observed for the addition of either GSH or ONOO^–^ separately. However, in the presence of both analytes, a clear fluorescence response was observed in live cells. **GSH-PF3** was further able to monitor the co-existence of metabolically produced ONOO^–^ and GSH by exogenous stimulation.

## Abstract 2

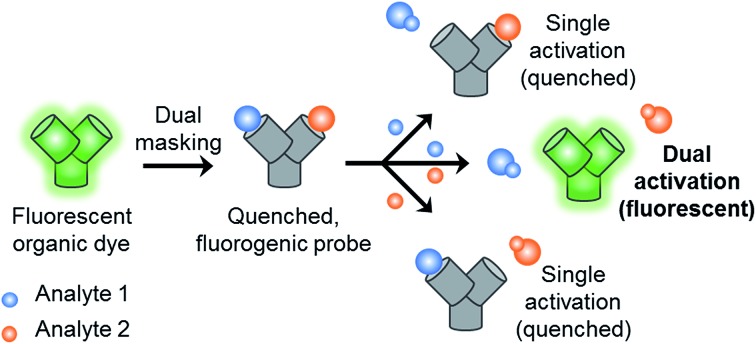
We have developed a novel AND logic based fluorescence probe for the simultaneous detection of ONOO^–^ and GSH (**GSH-PF3**).

Peroxynitrite (ONOO^–^), is a highly reactive nitrogen species that is formed *via* the diffusion controlled reaction between superoxide anion (O_2_˙^–^) and nitric oxide (NO˙).^1^,^2^ ONOO^–^ acts as a signalling molecule *in vivo* for a number of pathways.^1^,^3^ However, ONOO^–^ is more commonly known for its deleterious properties, causing irreversible damage to a range of biological targets such as lipids, proteins and DNA.^4^ Therefore, ONOO^–^ has been implicated as a key pathogenic factor for a number of diseases, which include inflammation, cancer, ischemia-reperfusion and neurodegenerative diseases.^5^–^7^ Glutathione (GSH) is a natural tripeptide (γ-l-glutamyl-l-cysteinyl-glycine), that exists in the thiol reduced form (GSH) and disulphide-oxidised (GSSG) form. GSH is the predominant form, existing at millimolar concentrations in most cells.^8^ However, GSH can be directly oxidised by ONOO^–^ therefore acting as a cellular defence by serving as an ONOO^–^ scavenger. Elevated levels of GSH are common in cells under oxidative stress and the susceptibility of a cell towards ONOO^–^ largely depends on the concentration of intracellular GSH.^1^,^9^,^10^

Therefore, with this work we set out to develop a fluorescence-based probe capable of monitoring the close relationship between ONOO^–^ and GSH. Traditionally, most fluorescence probes require a single analyte to produce a fluorescence response.^11^–^19^ However, in recent years a number of fluorescence based probes for dual or multi-analyte detection have been developed.^20^–^27^ These types of fluorescence based probes have been used for the construction of molecular logic gates or for medical diagnostics.^28^ AND logic based fluorescence probes require both analytes to be simultaneously present or work in tandem in order to elicit a fluorescence response. This method has a number of advantages including: being faster than serial measurements for different analytes within the same biological sample and can provide a method for monitoring bimolecular events, which may contribute to a specific disease.^20^

Currently, only a few reversible fluorescence based probes for the detection of ONOO^–^ and GSH have been developed to monitor the relationship between these analytes.^29^,^30^ These include a selenium based fluorescence probe, which is oxidised by ONOO^–^ (turn “on”) and reduced by GSH (turn “off”). The fluorescence of the CyPSe probe is initially quenched by a photoinduced electron transfer (PET) process. The presence of ONOO^–^ results in the oxidation of selenium to CyPSe=O causing the fluorescence emission to be “turned on”. Then in the presence of biological thiols such as cysteine and GSH, the CyPSe=O probe is reduced back to its non-fluorescent selenide form. A similar reversible NIR Tellurium-based fluorescence probe was later developed for monitoring the redox cycles between ONOO^–^ and GSH. This probe was successfully applied for the visualisation of the redox cycles of ONOO^–^ and GSH in live cells and animals.^29^ The probes developed by Han *et al.* are turn “on” with ONOO^–^ and “off” with GSH. As far as we are aware, there are currently no AND logic based fluorescence probes for GSH “and” ONOO^–^.

With this research, we aimed to develop a GSH “and” ONOO^–^ logic based fluorescence probe (Scheme 1). We identified a suitable mono-boronate fluorescein based fluorescence probe, **PF3** that had been previously developed by Chang *et. al.*, shown in Scheme 2. As previously reported the reactivity of boronate based fluorescence probes with ONOO^–^,^12^,^14^,^15^ are significantly greater than hypochlorite (ClO^–^) and H_2_O_2_.^31^ Therefore, we anticipated that the attachment of a GSH reactive motif to **PF3** would produce a selective GSH-ONOO^–^ AND logic based fluorescence probe, **GSH-PF3** (Scheme 2). **GSH-PF3** was readily synthesised in three steps. Fluorescein was triflated using *N*-phenyl bis(trifluoromethanesulfonamide) to afford fluorescein mono-triflate in good yield. Suzuki–Miyaura conditions were then carried out to provide fluorescein mono-boronate, **PF3**. The 2,4-dinitrobenzenesulfonyl unit was then attached to **PF3** using 2,4-dinitrobenzenesulfonyl chloride, CH_2_Cl_2_ and NEt_3_ at 0 °C. Using these conditions **GSH-PF3** was prepared in a reasonable yield of 52%.

**Scheme 1:**
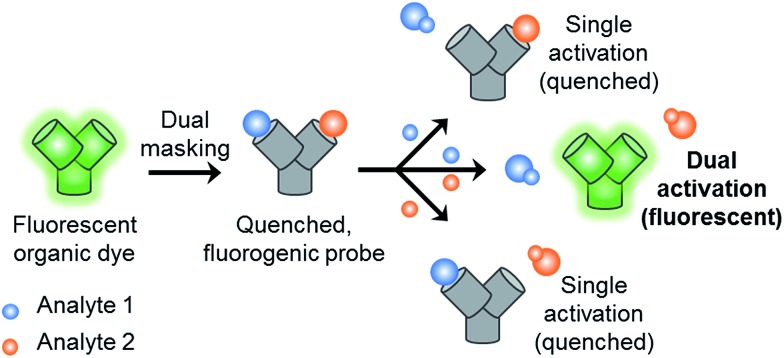
Design concept for AND logic based fluorogenic probe. A fluorescence dye is masked by two functional groups, which respond to two different analytes. The fluorogenic probe requires both analytes to be present or to work in tandem in order to produce a response.

**Scheme 2:**
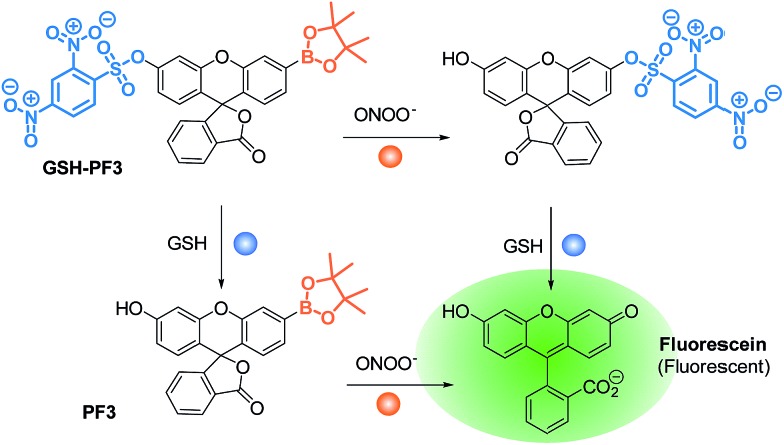
Structure of the **GSH-PF3** probe and proposed sensing mechanism for the simultaneous detection of ONOO^–^ and GSH.

With **GSH-PF3** in hand, fluorescence experiments for the detection of GSH and ONOO^–^ were performed. As shown in Fig. 1, **GSH-PF3** was initially non-fluorescent and with the addition of ONOO^–^ (10 μM), a small fluorescence increase was observed. However, incremental additions of GSH resulted in a much larger increase in fluorescence intensity (>30-fold see ESI – Fig. S1†), clearly demonstrating the need for the addition of both GSH and ONOO^–^ in order to achieve a full ‘turn-on’ response.

**Fig. 1:**
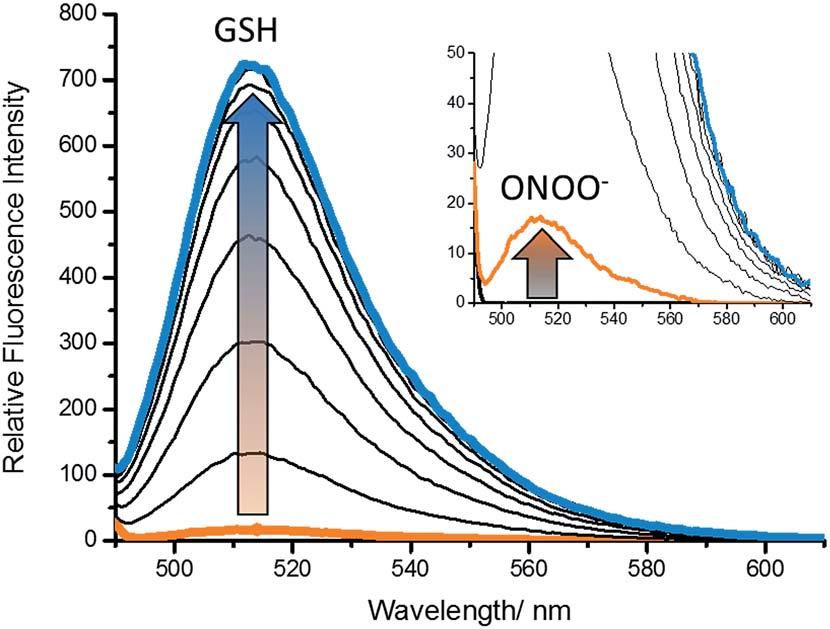
Fluorescence spectra of **GSH-PF3** (0.5 μM) with addition of ONOO^–^ (10 μM) (inset) followed by the addition of GSH (0–80 μM), 5 min wait between addition in buffer solution [52 wt% methanol] (pH = 8.21 at 25 °C). Fluorescence intensities were measured with *λ*_ex_ = 488 nm with slit widths ex slit: 5 nm and em slit: 2.5 nm.

In order to demonstrate that **GSH-PF3** required both GSH and ONOO^–^ for a complete ‘turn-on’ response, the fluorescence experiments were performed the other way around. Therefore, an excess of GSH (200 μM) was added to **GSH-PF3**, and the probe was incubated for 10 min. Remarkably, this only led to a small increase in fluorescence intensity and the subsequent additions of ONOO^–^ resulted in a large fluorescence increase (Fig. 2 and ESI – Fig S2†). These results confirm that the probe requires both GSH and ONOO^–^ for a full fluorescence ‘turn-on’ response.

**Fig. 2:**
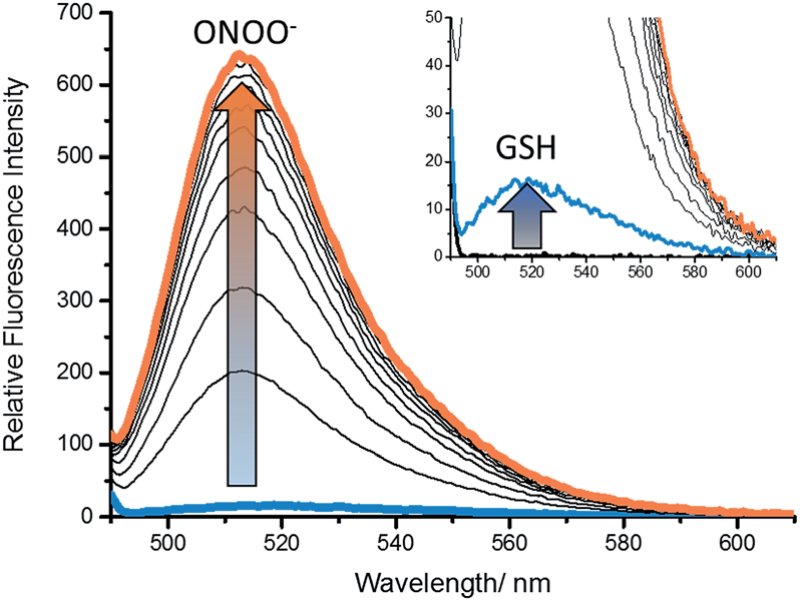
Fluorescence spectra of **GSH-PF3** (0.5 μM) with addition of GSH (200 μM), 10 min wait (inset), then addition of ONOO^–^ (0–10 μM) in buffer solution [52 wt% methanol] (pH = 8.21 at 25 °C). Fluorescence intensities were measured with *λ*_ex_ = 488 nm with slit widths ex slit: 5 nm and em slit: 2.5 nm.

The selectivity of **GSH-PF3** was then evaluated against a series of amino acids (l-cysteine, l-methionine, l-tryptophan, l-serine, l-lysine, l-leucine, l-glutamic acid, l-valine, l-arginine, l-histidine and l-aspartic acid) – see ESI Fig. S3.† Unsurprisingly, the probe responded to the other sulfhydryl containing amino acid cysteine. However, the biological concentrations of cysteine are low in cells.^32^ More importantly, **GSH-PF3** displayed an excellent selectivity against other amino acids including serine, methionine and lysine. The probe demonstrated excellent selectivity for ONOO^–^ over many other ROS including H_2_O_2_ (see ESI – Fig. S4†). The excellent selectivity for both GSH and ONOO^–^ allowed us to evaluate **GSH-PF3** in cell imaging experiments.

The macrophage cell line – RAW264.7 (ATCC® TIB-71™; obtained from ATCC [American Type Culture Collection]) – was used for cell imaging experiments. The cells were incubated with **GSH-PF3**, followed by either treatment of SIN-1 (an ONOO^–^ donor)^33^ to produce intracellular ONOO^–^ or by addition of exogenous GSH. As shown below in Fig. 3, addition of GSH separately resulted in no fluorescence response, whereas ONOO^–^ led to a small fluorescence response in cells. This observation is believed to be due to the presence of a low levels of endogenous biological thiols in the cells, resulting in the activation of the probe's fluorescence. As predicted, treatment of cells with both GSH and SIN-1 led to a clear fluorescence increase in RAW264.7 cells with **GSH-PF3**, in clear agreement with the analytical data obtained on the fluorimeter.

**Fig. 3:**
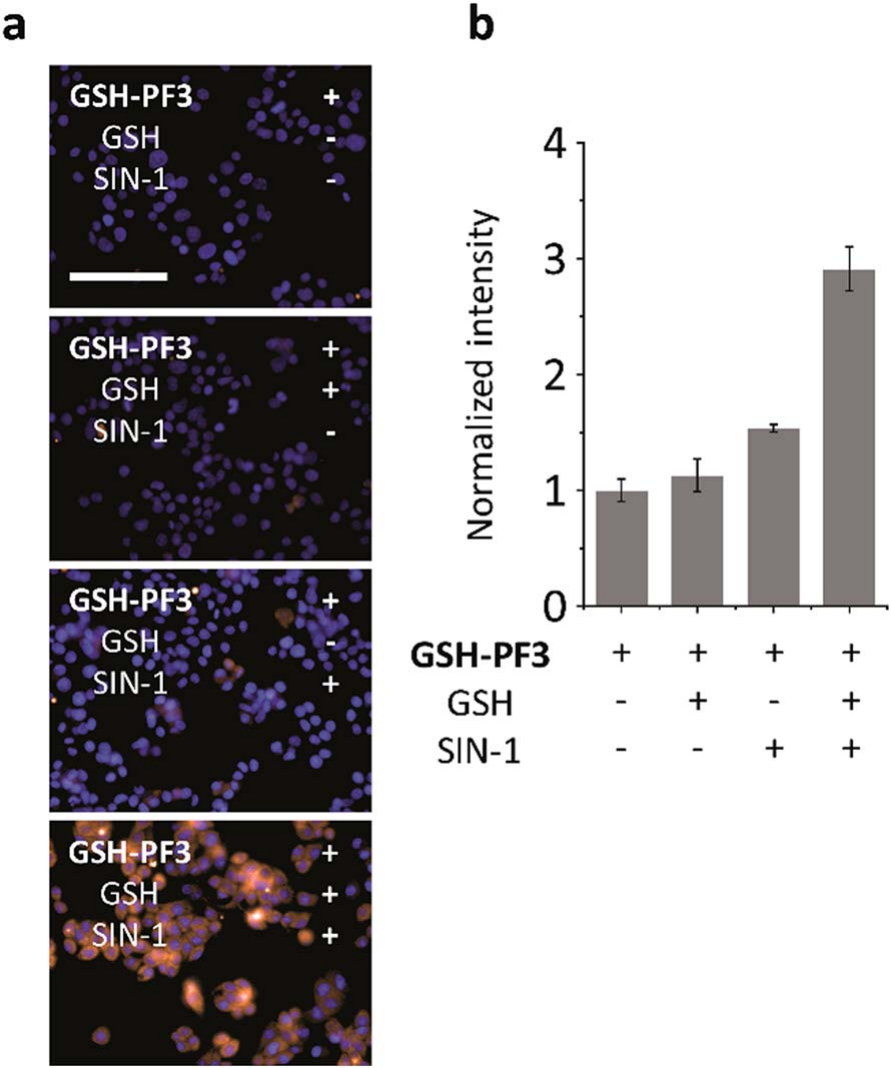
Fluorescence imaging (a) and quantification (b) of RAW264.7 cells with **GSH-PF3** (10 μM) in the absence and presence of exogenously added GSH (50 μM) and/or SIN-1 (500 μM). Excitation channel = 460–490 nm, emission channel filtered = 530–590 nm. Cell nuclei were stained with Hoechst 33342. Scale bar = 100 μm. Error bars represent SD.

We then turned our attention to evaluate the ability of **GSH-PF3** for monitoring the co-existence of metabolically produced ONOO^–^ by lipopolysaccharide (LPS) simulation^34^ and GSH through a drug treatment.^35^ It has been reported that LPS can elicit ONOO^–^ in macrophage, which is a signature of inflammation, whereas caffeic acid (CA) is commonly used to treat inflammation through the augmentation of intracellular GSH. Consequently, treatment with LPS, followed by the addition of increasing CA (0–100 μM) led to a gradual enhancement in the fluorescence intensity of **GSH-PF3** (Fig. 4). This result is due to the co-existence of ONOO^–^ produced by LPS stimulation as well as GSH elicited by CA. Interestingly, a further increase of the CA concentration (>100 μM) led to the suppression of probe's fluorescence, which is believed to be due to the production of an excess of GSH, resulting in quenching of the ONOO^–^ (Fig. 4). To corroborate the presence of intracellular GSH in macrophage, a commercial GSH probe was used. The result suggested that both the exogenous addition of GSH and stimulation of metabolic GSH by treatment of CA/LPS activated the commercial probe's fluorescence (Fig. S5†). Furthermore, a cell proliferation assay indicated that **GSH-PF3** is not toxic for RAW264.7 cells (Fig. S6†).

**Fig. 4:**
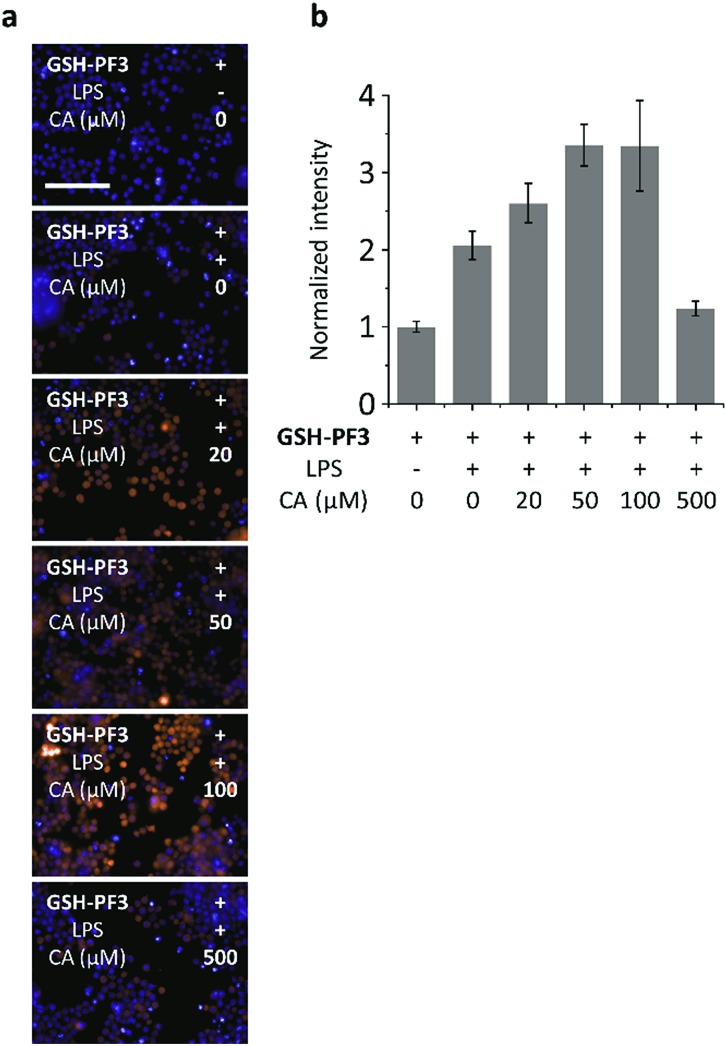
Fluorescence imaging (a) and quantification (b) of RAW264.7 cells with **GSH-PF3** (10 μM) in the absence and presence of LPS (1 μg mL^–1^), which elicits ONOO^–^ and increasing caffeic acid (CA, a drug that elicits endogenous GSH to quench ONOO^–^). Excitation channel = 460–490 nm, emission channel filtered = 530–590 nm. Cell nuclei were stained with Hoechst 33342. Scale bar = 100 μm. Error bars represent SD.

These results clearly demonstrate the potential of AND logic based fluorescence imaging probes for optimizing drug dosage in the treatment of inflammation and various other diseases. In addition this system could be used to help monitor the treatment of Alzheimer's disease (AD), since it is known that high levels of ONOO^–^ correlate with oxidative stress and the progression of AD, while, high levels of GSH have been implicated in AD therapy.^36^–^39^ Therefore, we believe that our **GSH-PF3** probe, which can detect GSH “and” ONOO^–^ could potentially be used to monitor cellular resistance towards ROS and map therapeutic improvements in AD victims.

In summary, **GSH-PF3** is an easy-to-prepare fluorescence based probe providing a platform for the development of other novel AND logic based fluorescence imaging probes for use in medical diagnostics. We are currently exploring the use of 6-amino/carboxyfluorescein, which we believe could provide the opportunity to attach additional fluorophores in order to develop ratiometric fluorescence sensors^40^ or for the attachment of targeting or therapeutic units.^41^–^43^

## Conflicts of interest

No conflicts of interest.

## Supplementary Material

Supplementary information
