# Supplementary material for: The development of a novel AND logic based fluorescence probe for the detection of peroxynitrite and GSH
Source: Chem Sci. 2018 Mar 16;9(15):3672–6. doi: 10.1039/c8sc00733k (PMC5935063; doi:10.1039/c8sc00733k)

Electronic Supplementary Material (ESI) for Chemical Science.
This journal is © The Royal Society of Chemistry 2018

Supplementary Information

The development of a novel AND logic based fluorescence probe for the detection of peroxynitrite and GSH

Adam C. Sedgwick,[a1]* Hai-Hao Han,[b1] Jordan E. Gardiner,[a] Steven D. Bull,[a]* Xiao-Peng He[b]* and Tony D. James[a]*

[a]Department of Chemistry, University of Bath, Bath, BA2 7AY, U.K.

[b]Key Laboratory for Advanced Materials and Joint International Research Laboratory of Precision Chemistry and Molecular Engineering, Feringa Nobel Prize Scientist Joint Research Center, School of Chemistry and Molecular Engineering, East China University of Science and Technology, 130 Meilong Rd., Shanghai 200237, China.
[1]Equal contribution

Email addresses.
A.C.Sedgwick@bath.ac.uk (ACS)
S.D.Bull@bath.ac.uk (SDB)
xphe@ecust.edu.cn (XPH)
T.D.James@bath.ac.uk (TDJ)

Content

1. **Fluorescence analysis**
2. **Cellular imaging methods**
3. **Experimental**
4. **NMR**

1. Fluorescence analysis

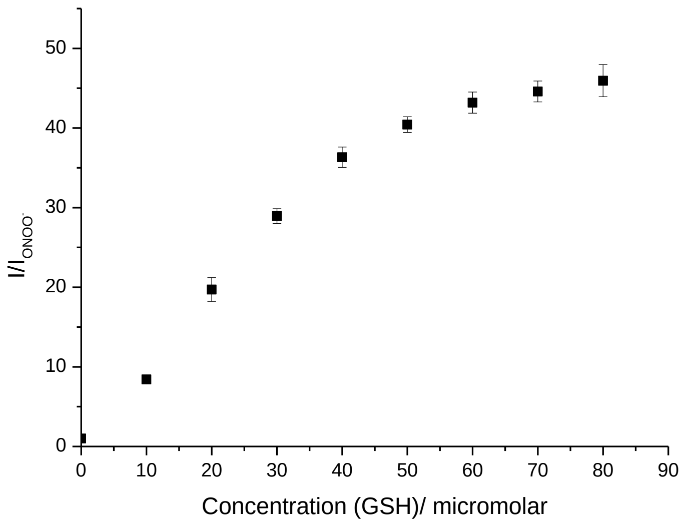

Fig. S1 - Fluorescence intensity changes (I/I_{ONOO^-}) for **GSH-PF3** (0.5 μM) with addition of $ONOO^-$ (10 μM) followed by the addition of GSH (0 - 80 μM), 5 min wait between addition in buffer solution [52 wt% methanol]. pH = 8.21 at 25 °C. Fluorescence intensities were measured with λ_{ex} = 488 nm/ λ_{em} = 512 nm with slit widths Ex slit: 5 nm and Em slit: 2.5 nm

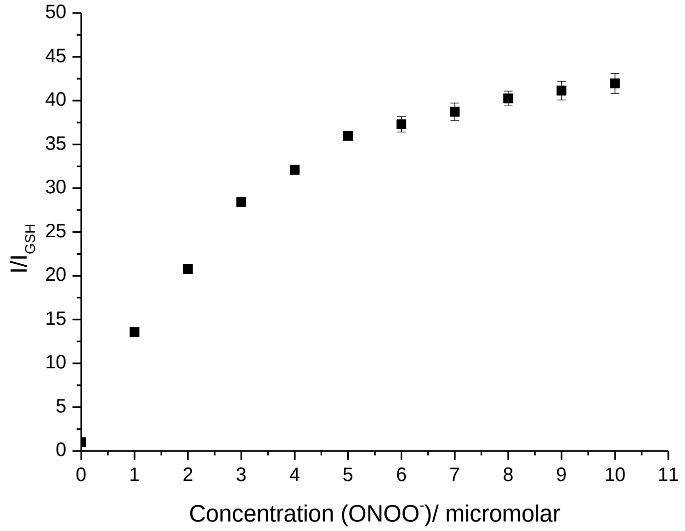

Fig. S2 - Fluorescence intensity changes (I/I_{GSH}) for **GSH-PF3** (0.5 μM with addition of GSH (200 μM) wait 10 min then addition of $ONOO^-$ (0 - 10 μM) in buffer solution [52 wt% methanol], pH = 8.21 at 25 °C. Fluorescence intensities were measured with λ_{ex} = 488 nm/ λ_{em} = 512 nm with slit widths Ex slit: 5 nm and Em slit: 2.5 nm

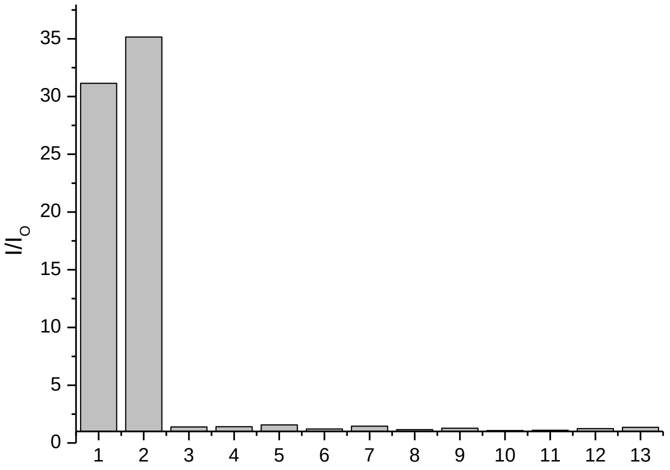

Fig. S3 - Selectivity bar chart of **GSH-PF3** (0.5 µM) with addition of $ONOO^-$ (10 µM) then addition of various amino acids - 100 µM (1- GSH, 2 – Cys, 3 – Met, 4 – Tryp, 5 – Ser, 6 – Lys, 7 – Leu, 8 – Glu, 9 – Val, 10 – Arg, 11 – His, 12 – Asp, 13 – Blank). 5 min wait before measurement in buffer solution [52 wt% methanol]. pH = 8.21 at 25 ^{o}C. Fluorescence intensities were measured with λ_{ex} = 488 nm/ λ_{em} = 512 nm with Slit Widths Ex slit: 5 nm and Em slit: 2.5 nm.

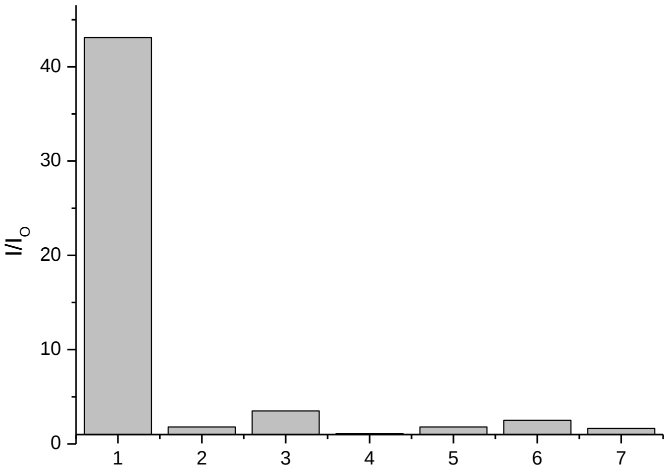

Fig. S4 - Selectivity bar chart of **GSH-PF3** (0.5 µM) with addition of GSH (200 µM) wait 10 min then addition of various ROS 100 µM (1 – $ONOO^-$ (10 µM), 2 – H_2O_2, 3 – ClO^-, 4 – KO_2, 5 – $^1O_2$, 6 – $HO^·$, 7 – ROO.) in buffer solution [52 wt% methanol], pH = 8.21 at 25 ^{o}C. Fluorescence intensities were measured with λ_{ex} = 488 nm\ λ_{em} = 512 nm with Slit Widths Ex slit: 5 nm and Em slit: 2.5 nm

3. Cellular experiments

Cell culture. RAW 264.7 cells were maintained in a Dulbecco's Modified Eagle's Medium (Invitrogen, Carlsbad, CA, USA) supplemented with 10% fetal bovine serum (Gibco, Gland Island, NY, USA) in a humidified atmosphere of 5% CO_2 and 95% air at 37 ºC and split when the cells reached 90% confluency.

Fluorescence imaging of cells. Cells were seeded on a black 96-well microplate with optically clear bottom (Greiner bio-one, Germany) overnight. For SIN-1/GSH imaging experiments, the cells were incubated with **GSH-PF3** (10 µM, dissolved in PBS containing 1% DMSO, pH 7.4) for 30 min, followed by incubation with SIN-1 (500 µM), or GSH (50 µm), or the mixture of SIN-1 (500 µM) and GSH (50 µm) for 30 min. For LPS/CA imaging experiments, caffeic acid was diluted with PBS and treated with the cell cultures at final concentrations of 0, 20, 50, 100 and 500 µM alone or with 1 µg/mL of LPS for a day. After 24-h incubation, cells were incubated with **GSH-PF3** (10 µM, dissolved in PBS containing 1% DMSO, pH 7.4) for 20 min. The cells nuclei were stained with Hoechst 33342 (5 µg mL^{-1}) at 37 °C in a humidified atmosphere of 5% CO_2 in air for 5 min. Then, cells were washed with PBS three times. The fluorescence images were recorded using an Operetta high-content imaging system (Perkinelmer, US), and was quantified and plotted by Columbus analysis system (Perkinelmer, US).

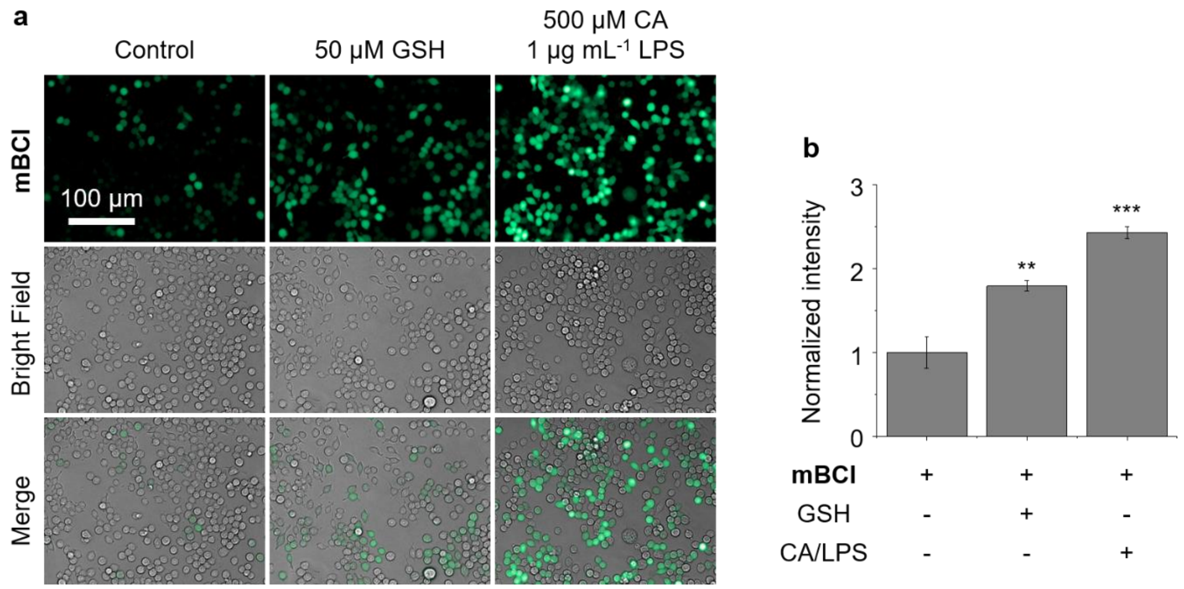

Fig. S5 - Fluorescence imaging (a) and quantification (b) of RAW264.7 cells with **mBCl** (monochlorobimane, a commercial GSH dye; 20 µM) with or without subsequent addition of GSH (50 µM) or treatment with caffeic acid (CA; 500 µM) and lipopolysaccharide (LPS; 1 µg mL^{-1}) (***P*< 0.01, ****P*< 0.005). Excitation channel = 360–400 nm, emission channel filtered = 460–540 nm. Error bars represent S. D.

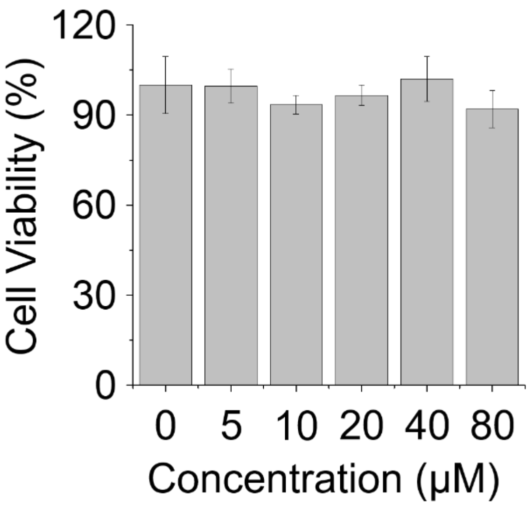

Fig. S6 - Cell viability of RAW264.7 with increasing **GSH-PF3**. Cells were plated overnight on 96-well plates in growth medium. After seeding, cells were treated with **GSH-PF3** of different concentrations for 24 hours. Then, a solution of MTS/PMS (20:1, Promega Corp) (10 μL per well) was added to each well containing 100 μL of growth medium. After incubation at 37°C under 5% CO_2 for 2 h, the absorbance of the solutions was measured at 490 nm using an M5 microplate reader (Molecular Device, USA). The optical density of the result in MTS assay was directly proportional to the number of viable cells.

4. Experimental

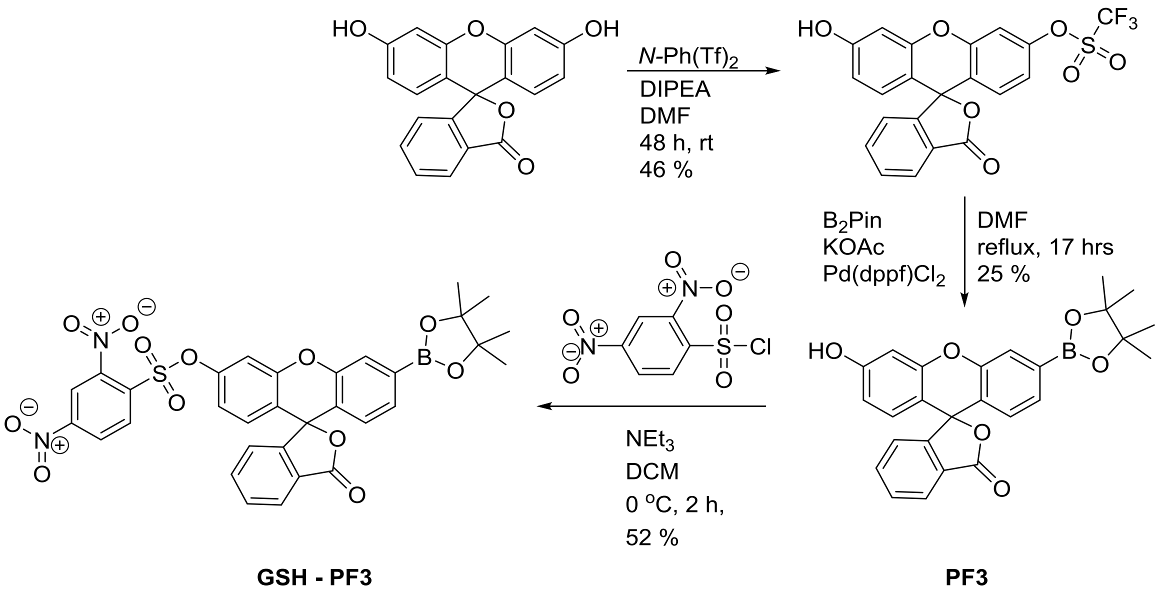

3'-hydroxy-3-oxo-3H-spiro[isobenzofuran-1,9'-xanthen]-6'-yl trifluoromethanesulfonate

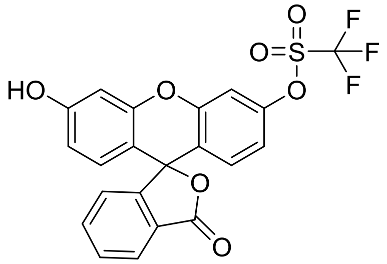

Following the literature procedure[28], Fluorescein (2.00 g, 5.8 mmol) and *N*-Phenyl bis(trifluoromethanesulfonamide) (2.1g, 5.8 mmol) was dissolved in dry DMF (15 mL) and flushed with nitrogen. Diisopropylethylamine (3.8 mL) was then added and the reaction mixture was stirred for 48 h. The reaction mixture was quenched with 1 M HCl (100 mL) and extracted with EtOAc (3 x 100 mL). The combined organics were dried ($MgSO_4$) and concentrated *in-vacuo* to afford the crude material. The crude was purified *via* column chromatography EtOAc/Pet Ether (10:90 to 50:50) and the title compound was isolated as a white solid (1.24 g, 2.67 mmol, 46 %). M.P. – 139 – 142 ^oC; ^1H NMR (300 MHz, DMSO-d_6) d 10.30 (br. s., 1 H, O-H), 8.05 (d, *J* = 7.0 Hz, 1 H, Ar*H*), 7.87 - 7.67 (m, 4 H, Ar*H*), 7.38 (d, *J* = 7.5 Hz, 1 H, Ar*H*), 7.24 (dd, *J* = 2.6, 8.9 Hz, 1 H, Ar*H*), 7.02 (d, *J* = 8.9 Hz, 1 H, Ar*H*), 6.78 - 6.72 (m, 1 H, Ar*H*), 6.63 (s, 2 H, Ar*H*); ^{13}C NMR (75.5 MHz, DMSO-d_6) d 168.8, 160.2, 152.4, 151.7, 151.6, 150.0, 136.3, 130.9, 129.6, 125.9, 125.3, 124.5, 120.7, 120.2, 117.6, 116.4, 113.8, 111.1, 109.1, 102.6, 81.6, 60.1; I.R (thinfilm) ν max (cm^{-1}): 3389.62 (O-H), 1736.19 (C=O); HRMS (TOF MS ASAP+): m/z calculated for $C_{21}H_{11}F_3O_7S$: requires 465.0256 for $[M+H]^+$, found 465.0256

3'-Hydroxy-6'-(4,4,5,5-tetramethyl-1,3,2-dioxaborolan-2-yl)-3H-spiro[isobenzofuran-1,9'-xanthen]-3-one

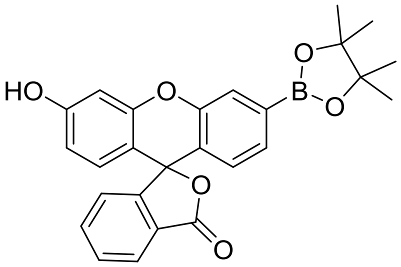

To a solution of 3'-Hydroxy-3-oxo-3H-spiro[isobenzofuran-1,9'-xanthen]-6'-yl trifluoromethanesulfonate (0.630 g, 1.36 mmol) in DMF (10 mL). Bis(pinacolato) diboron (0.62 g, 2.44 mmol), KOAc (0.80 g, 8.15 mmol) and Pd(dppf)Cl_2.DCM (0.10 g, 0.14 mmol) was added and the reaction mixture was heated at 95 ^oC under N_2 for 16 h. The reaction mixture was then cooled to rt and EtOAc (100 mL) and H_2O (100 mL) were added. The aqueous layer was extracted with EtOAc (2 x 100 mL). The combined organics were then washed with H_2O (2 x 100 mL), brine (100 mL) and dried ($MgSO_4$) and concentrated *in-vacuo* to afford the crude material. The crude material was purified *via* column chromatography EtOAc:Pet Ether (10:90 to 40:60) to afford the title compound as a pale clear oil (0.15 g, 0.34 mmol, 25 %). ^{1}H NMR (500 MHz, $CDCl_3$) 8.04 (d, *J* = 7.3 Hz, 1 H), 7.73 (s, 1 H), 7.68 - 7.59 (m, 2 H), 7.43 (d, *J* = 7.8 Hz, 1 H), 7.14 (d, *J* = 7.3 Hz, 1 H), 6.81 - 6.76 (m, 2 H), 6.65 (d, *J* = 8.8 Hz, 1 H), 6.56 (dd, *J* = 2.2, 8.6 Hz, 1 H), 1.36 (s, 12 H); ^{13}C NMR (125.75 MHz, $CDCl_3$) 170.0, 158.2, 153.4, 152.4, 150.7, 135.2, 129.8, 129.3, 129.2, 127.2, 126.3, 125.1, 123.9, 123.5, 121.4, 112.4, 110.6, 103.3, 84.3, 24.8, 24.8; I.R (thinfilm) ν max (cm^{-1}): 1746.51 (C=O); HRMS (TOF MS ASAP+): m/z calculated for $C_{26}H_{23}BO_6$: requires 442.1702 for $[M+H]^+$, found 442.1700

3-Oxo-3'-(4,4,5,5-tetramethyl-1,3,2-dioxaborolan-2-yl)-3H-spiro[isobenzofuran-1,9'-xanthen]-6'-yl 2,4-dinitrobenzenesulfonate

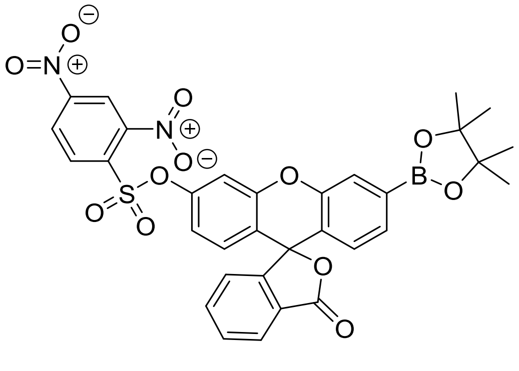

2,4-Dinitrobenzenesulfonyl chloride (0.089 g, 0.033 mmol) in DCM (3 mL) was added dropwise to a solution of 3'-hydroxy-6'-(4,4,5,5-tetramethyl-1,3,2-dioxaborolan-2-yl)-3H-spiro[isobenzofuran-1,9'-xanthen]-3-one (0.15 g, 0.34 mmol) and NEt_3 (95 µL, 0.69 mmol) in DCM (5 mL) at 0 ^{o}C. The reaction mixture was stirred at 0 ^{o}C for 2 h before the addition of H_2O (15 mL) and DCM (15 mL). The organic layer was washed with H_2O (2 x 10 mL), brine (10 mL) and dried ($MgSO_4$) and concentrated *in-vacuo* to afford the crude material. The crude material was purified *via* column chromatography EtOAc:Pet Ether (10:90 to 40:60) to afford the title compound as a pale yellow solid (0.115 g, 0.17 mmol, 52 %); M.p. 119-122 ^{o}C; ^{1}H NMR (500MHz, $CDCl_3$) 8.67 (d, *J* = 2.0 Hz, 1 H, Ar*H*), 8.53 (dd, *J* = 2.0, 8.8 Hz, 1 H, Ar*H*), 8.25 (d, *J* = 8.8 Hz, 1 H, Ar*H*), 8.03 (d, *J* = 7.3 Hz, 1 H, Ar*H*), 7.73 (s, 1 H, Ar*H*), 7.69 - 7.62 (m, 2 H, Ar*H*), 7.47 (d, *J* = 7.8 Hz, 1 H, Ar*H*), 7.19 (d, *J* = 2.0 Hz, 1 H, Ar*H*), 7.11 (d, *J* = 7.8 Hz, 1 H, Ar*H*), 6.91 (dd, *J* = 2.2, 8.6 Hz, 1 H, Ar*H*), 6.86 (d, *J* = 8.3 Hz, 1 H, Ar*H*), 6.82 (d, *J* = 7.8 Hz, 1 H, Ar*H*), 1.35 (s, 12 H, B*Pin*); ^{13}C NMR (125.75 MHz, $CDCl_3$) 169.0, 153.0, 151.9, 151.1, 150.0, 149.5, 149.0, 135.4, 134.0, 133.3, 130.2, 130.2, 129.8, 127.0, 126.7, 125.6, 125.4, 123.7, 123.4, 121.0, 120.5, 119.2, 117.2, 111.0, 84.3, 24.9, 24.8, 24.8; I.R (thinfilm) ν max (cm^{-1}): 1766.44 (C=O); HRMS (FTMS-NSI): m/z calculated for $C_{32}H_{25}BN_2O_{12}S$: requires 672.1330 for $[M+H]^+$, found 672.1329

5. NMR

3'-Hydroxy-3-oxo-3H-spiro[isobenzofuran-1,9'-xanthen]-6'-yl trifluoromethanesulfonate (300 MHz, DMSO-d6)

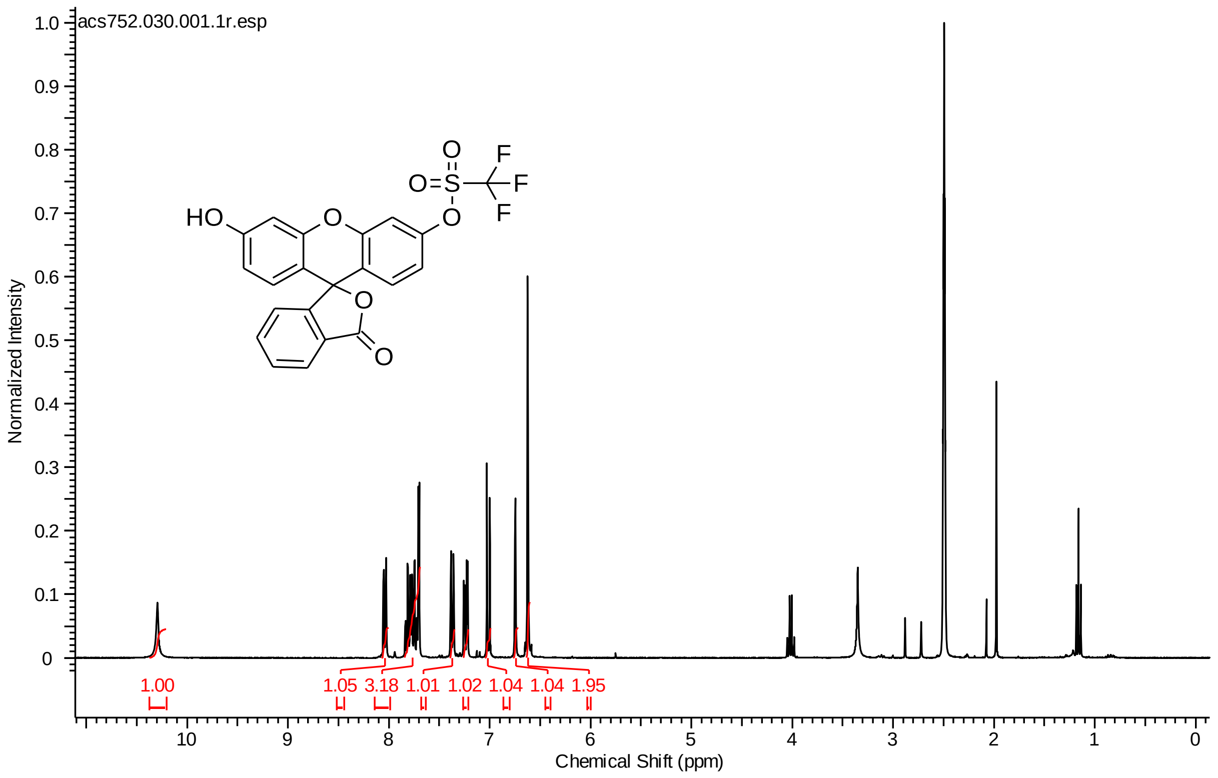

3'-Hydroxy-3-oxo-3H-spiro[isobenzofuran-1,9'-xanthen]-6'-yl trifluoromethanesulfonate

(75.5 MHz, DMSO-d6)

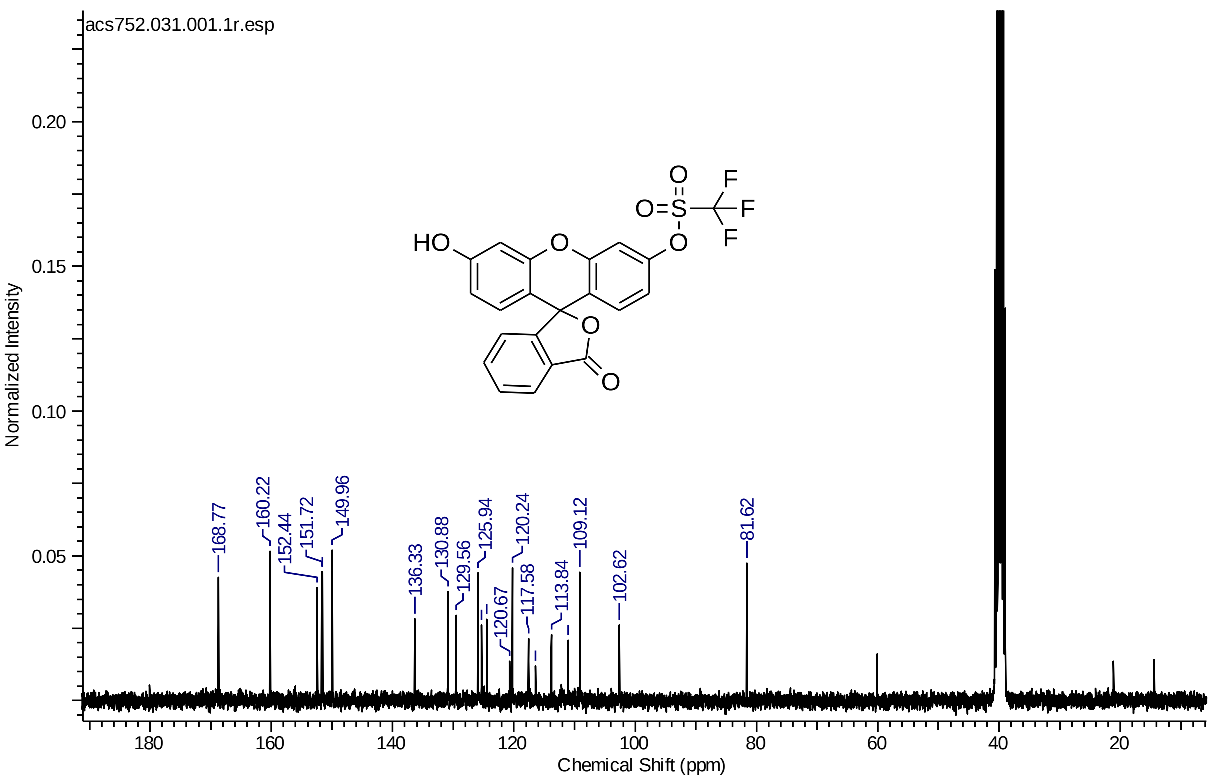

3'-Hydroxy-6'-(4,4,5,5-tetramethyl-1,3,2-dioxaborolan-2-yl)-3H-spiro[isobenzofuran-1,9'-xanthen]-3-one (500 MHz, $CDCl_3$)

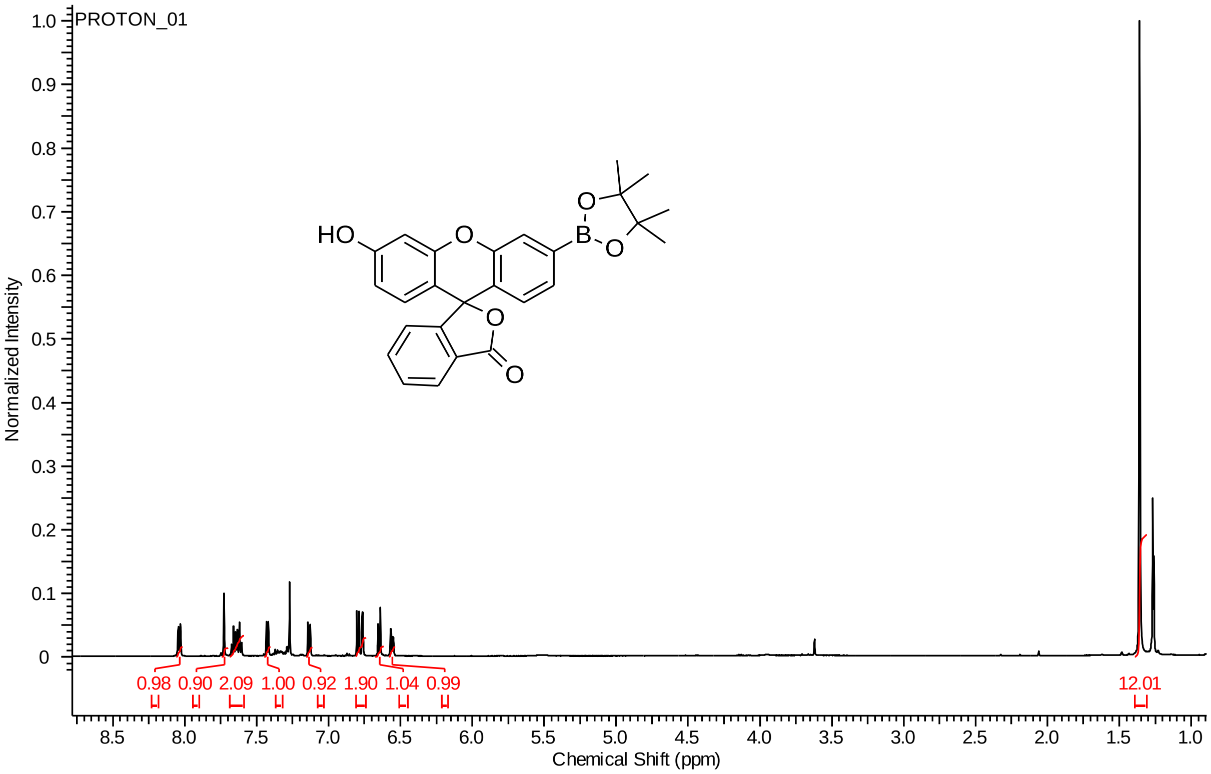

3'-Hydroxy-6'-(4,4,5,5-tetramethyl-1,3,2-dioxaborolan-2-yl)-3H-spiro[isobenzofuran-1,9'-xanthen]-3-one (125.75 MHz, $CDCl_3$)

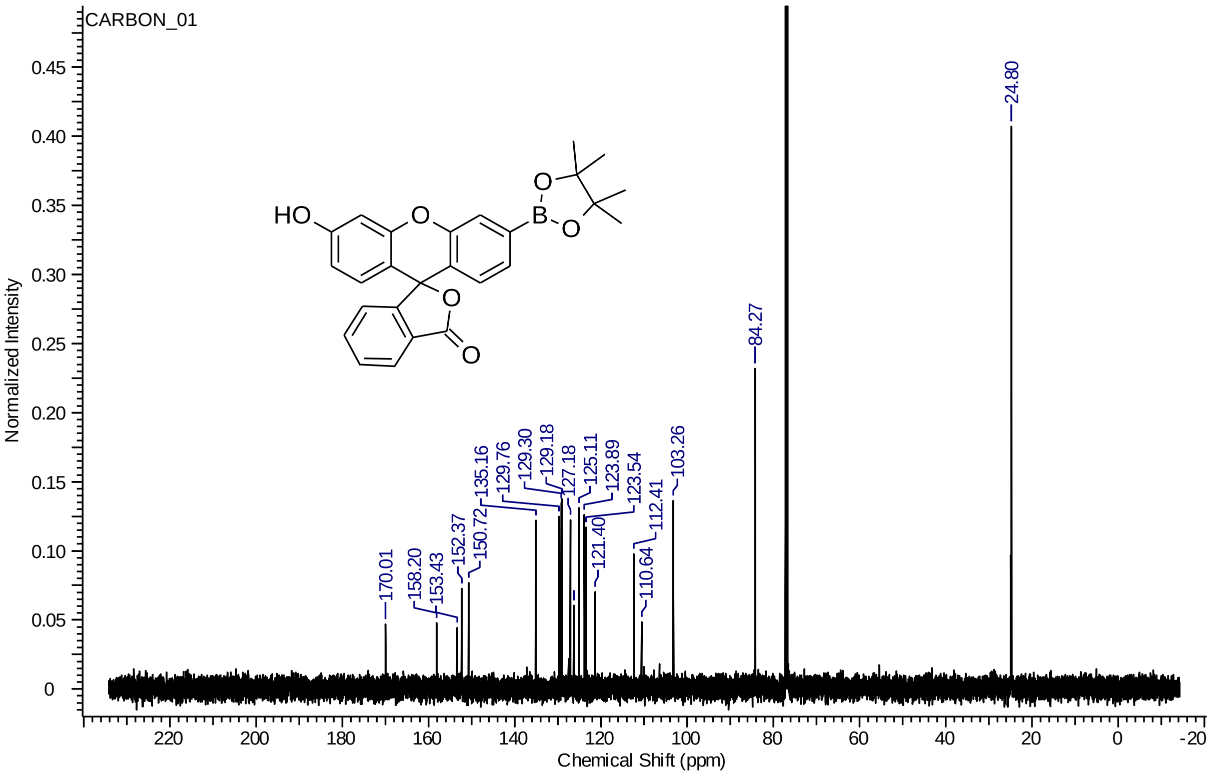

3-Oxo-3'-(4,4,5,5-tetramethyl-1,3,2-dioxaborolan-2-yl)-3H-spiro[isobenzofuran-1,9'-xanthen]-6'-yl 2,4-dinitrobenzenesulfonate (500 MHz, $CDCl_3$)

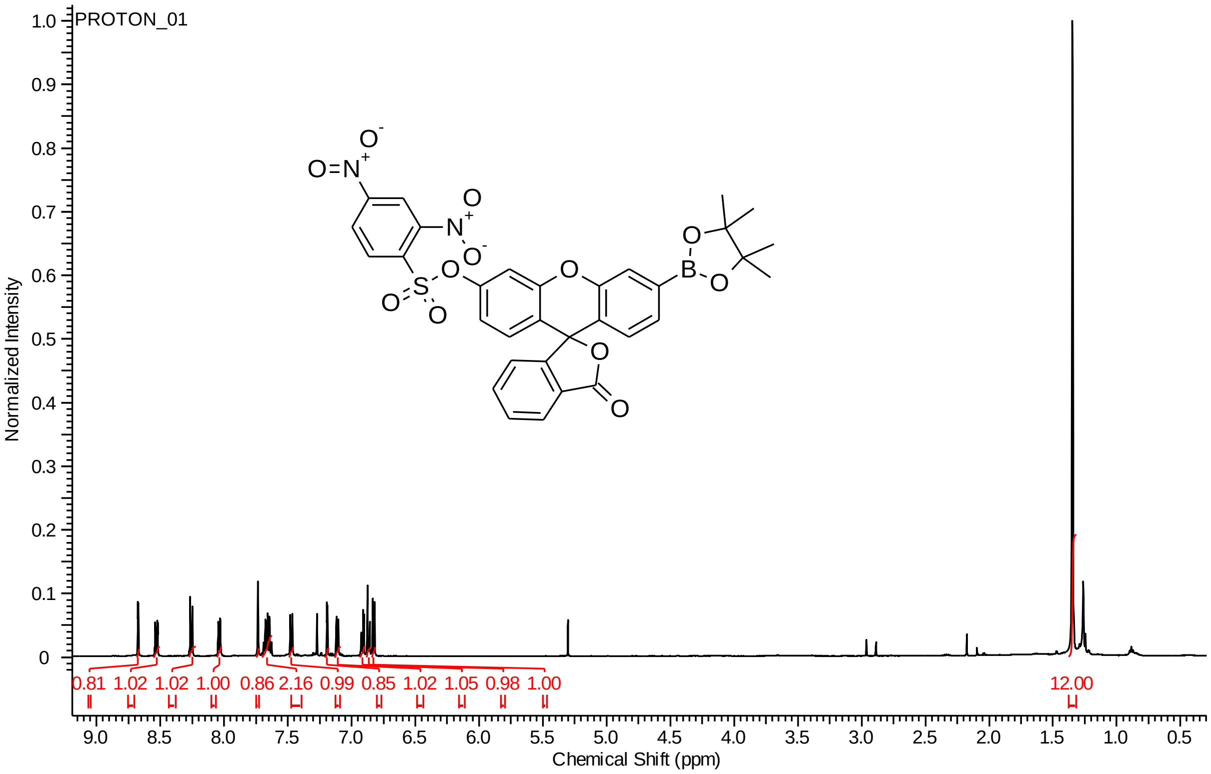

3-Oxo-3'-(4,4,5,5-tetramethyl-1,3,2-dioxaborolan-2-yl)-3H-spiro[isobenzofuran-1,9'-xanthen]-6'-yl 2,4-dinitrobenzenesulfonate (125.75 MHz, $CDCl_3$)

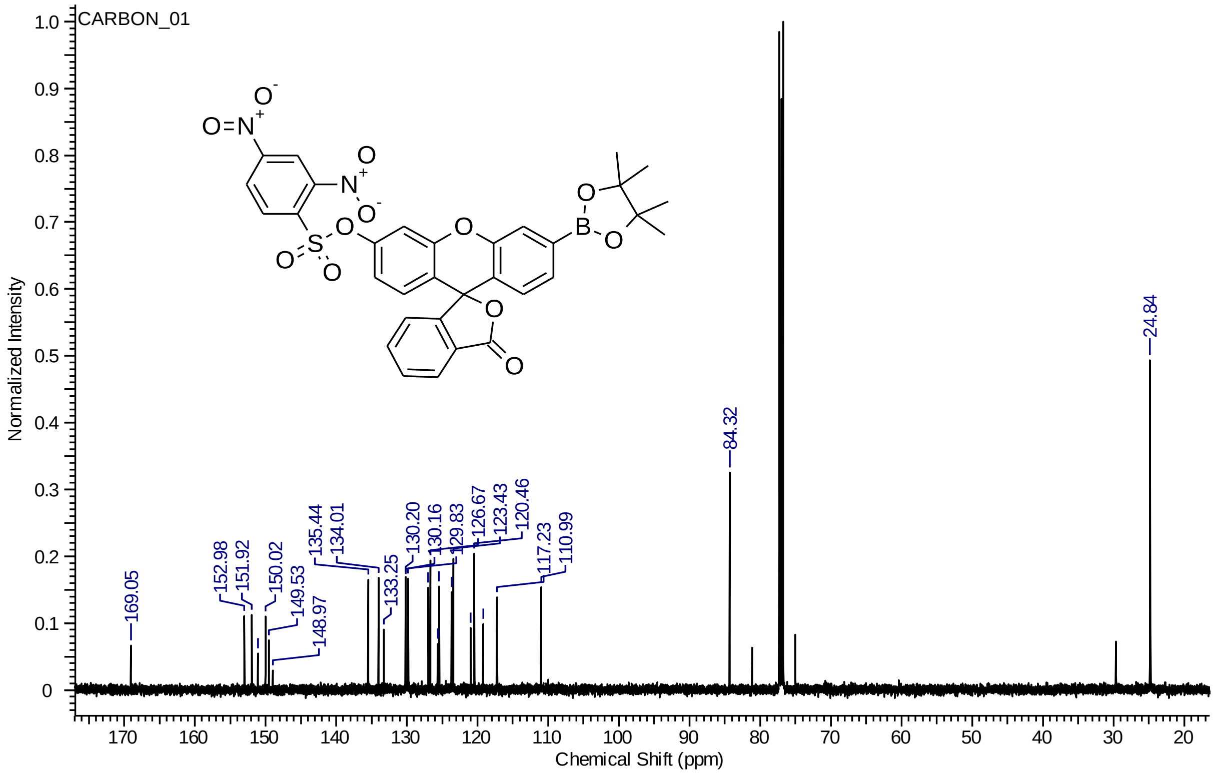

Supplement: Supplementary file 1 [file SC-009-C8SC00733K-s001.pdf]
